# Prevalence of suspected bruxism and its association with anxiety, depression, and stress among persons deprived of liberty in the Peruvian Amazon: a cross-sectional study

**DOI:** 10.3389/froh.2026.1949416

**Published:** 2026-09-29

**Authors:** Martín Jalk-Tiravanti, Alan Fernando Hernandez-Chuquimbalqui, Marvin Guevara-Vargas, Edwin Gonzales-Paco, Heber Isac Arbildo-Vega, Franz Tito Coronel-Zubiate

**Affiliations:** 1 Faculty of Health Sciences, Psychology School, Universidad Nacional Toribio Rodríguez de Mendoza de Amazonas, Chachapoyas, Peru; 2 Faculty of Health Sciences, Nursing School, Universidad Nacional Toribio Rodríguez de Mendoza de Amazonas, Chachapoyas, Peru; 3 Faculty of Dentistry, Dentistry School, Universidad San Martín de Porres, Chiclayo, Peru; 4 Faculty of Human Medicine, Human Medicine School, Universidad San Martín de Porres, Chiclayo, Peru; 5 Graduate School, Universidad Nacional Toribio Rodríguez de Mendoza de Amazonas, Chachapoyas, Peru; 6 Faculty of Health Sciences, Stomatology School, Universidad Nacional Toribio Rodríguez de Mendoza de Amazonas, Chachapoyas, Peru

**Keywords:** anxiety, depression, oral health, persons deprived of liberty, Peru, stress, suspected bruxism

## Abstract

**Background:**

Suspected bruxism remains poorly documented in prison populations, particularly in Latin America, where the intersection between mental and oral health has been scarcely investigated.

**Objective:**

To determine the prevalence of suspected bruxism and examine its relationship with anxiety, depression, stress, and clinical oral health indicators among persons deprived of liberty in the Peruvian Amazon.

**Methods:**

A cross-sectional census included 201 eligible participants from three wards of the Huancas Penitentiary Establishment. Suspected bruxism was identified using the Clinical Self-Report Screening Test (McNeill, 1993; modified by Kindernecht, 1997), with a positive result defined as ≥2 affirmative responses across 25 items. Anxiety, depression, and stress were assessed using the DASS-21, and oral health using the DMFT index and Basic Erosive Wear Examination (BEWE). Bivariate analyses included Chi-square or Fisher's exact tests and Spearman correlations. Binary logistic regression was performed, with multicollinearity assessed using the Variance Inflation Factor (VIF) and model fit evaluated using the Hosmer-Lemeshow test and Nagelkerke R^2^.

**Results:**

Suspected bruxism prevalence was 72.6% (*n* = 146; 95% CI: 66.1–78.3%), with the highest prevalence observed in the women's ward (80.5%). Severe anxiety showed a significant bivariate association with suspected bruxism (11.6% vs. 0.0%; *p* = 0.028). Weak but statistically significant positive associations were observed between suspected bruxism and depression (rho=0.159; *p* < 0.05) and stress (rho=0.179; *p* < 0.05). No statistically significant independent associations were identified in the logistic regression (all *p* > 0.05), in the context of moderate multicollinearity (maximum VIF=7.10) and sparse observations in some categories. Among participants with available DMFT data, 81.1% had a high caries index, while mean BEWE was 8.00 ± 6.57, with no significant difference according to suspected bruxism status (*p* = 0.40).

**Conclusions:**

A high prevalence of screening-defined suspected bruxism was observed among persons deprived of liberty in the Peruvian Amazon. Severe anxiety showed a significant bivariate association, while depression and stress showed weak positive associations with suspected bruxism. These findings highlight the potential value of considering psychological and oral health jointly in correctional healthcare and warrant further research using standardized bruxism-specific clinical or instrumental assessment.

## 1 Introduction

Bruxism is a masticatory muscle activity that may involve teeth clenching or grinding and may occur during sleep (sleep bruxism) or while awake (awake bruxism). Its prevalence in the general adult population ranges from 8% to 31.4%, with the awake subtype being more frequent (22.1%–31%) compared to sleep bruxism (12.8%) (1–3). The etiology of bruxism is multifactorial, and among the most extensively studied triggering factors are psychological variables, particularly anxiety, stress, and depression (4–7). A recent meta-analysis confirmed that individuals experiencing stress have a 97% higher likelihood of presenting bruxism compared to those without stress (7).

Prison populations constitute one of the most vulnerable human groups in terms of mental health. Deprivation of liberty generates chronic conditions of psychosocial stress, overcrowding, loss of autonomy, and uncertainty about the future, which considerably increases the burden of mental disorders (8–11). An umbrella review published in The Lancet Public Health in 2024 confirmed that the prevalence of mental disorders among persons deprived of liberty is substantially higher than in the general population, with anxiety and depression disorders being the most common (11, 12). González-Riera et al. (2024) documented increases in stress, anxiety, and depression among prison inmates during periods of prolonged isolation (13).

Despite this elevated psychological burden, the intersection between mental health and suspected bruxism in prison contexts has been scarcely investigated. While studies on general oral health among inmates exist, evidence regarding the prevalence of suspected bruxism and its relationship with psychological symptoms in this population remains limited (14–16). In Peru, previous research conducted at the Huancas Penitentiary documented a generalized burden of untreated oral disease, particularly edentulism, and highlighted the limited availability of oral rehabilitation services within the prison system, underscoring the need for broader oral health research in incarcerated populations (17). Studies in India and Portugal have reported high prevalence of dental caries and periodontal disease among inmates, with a pooled caries prevalence of 78.4% according to a recent systematic review (18). However, bruxism as an oral health variable has barely been assessed in this population. The scoping review by Amaya et al. (2024) on oral health services in prisons worldwide identified bruxism among the dental problems present, but without consolidated prevalence data (19).

The Huancas Penitentiary Establishment (PE), located in the Amazonas region, one of the most remote areas of Peru, houses persons deprived of liberty with limited access to both dental and mental healthcare services. Although prison populations are recognized as experiencing a disproportionate burden of psychological disorders and poor oral health, no previous study has simultaneously evaluated suspected bruxism, psychological symptoms, and clinical oral health indicators among persons deprived of liberty in the Peruvian Amazon. Investigating these conditions together may provide a more comprehensive understanding of the interplay between psychological distress and oral health while characterizing their simultaneous burden in this highly vulnerable population. Such evidence may also inform future research on integrated approaches to oral and mental healthcare in correctional settings. Therefore, the present study aimed to determine the prevalence of suspected bruxism and examine its relationship with anxiety, depression, stress, and clinical oral health indicators (DMFT and BEWE) among persons deprived of liberty at the Huancas Penitentiary Establishment. We hypothesized that greater psychological distress would be associated with a higher prevalence of suspected bruxism and poorer oral health.

## 2 Material and methods

### 2.1 Study design

This was an observational, cross-sectional, and correlational study conducted in accordance with the STROBE reporting guidelines for cross-sectional studies (20). Statistical analysis was performed using R version 4.5.3.

### 2.2 Participants

The study population consisted of persons deprived of liberty from the Huancas Penitentiary Establishment (PE), Chachapoyas, Amazonas, Peru. All persons meeting the eligibility criteria were included (*n* = 201); therefore, sample size calculation was not performed because the study constituted a population census. Participants were distributed across three pavilions: Women (*n* = 41; 20.4%), Primary Regime (*n* = 73; 36.3%), and Minimum Security (*n* = 87; 43.3%).

Inclusion criteria: Persons deprived of liberty with more than 30 days of incarceration, without prior diagnosis of neurodegenerative disorder, with natural dentition present (minimum 8 teeth), and who provided verbal informed consent.

Exclusion criteria: Persons were excluded if they met any of the following conditions: active psychotropic pharmacological treatment (antidepressants, anxiolytics, or antipsychotics), given that these medications may influence mandibular muscle activity and potentially affect bruxism-related symptoms; fewer than eight natural teeth present, as clinical evaluation requires sufficient dental surfaces for oral assessment; prior diagnosis of neurodegenerative disorder, due to its potential interference with responses to self-report instruments; and failure to complete the assessment instruments.

### 2.3 Instrument

The 21-item Depression, Anxiety and Stress Scale (DASS-21) is a self-report instrument that assesses negative emotional symptomatology across three dimensions: depression, anxiety, and stress (21). The scale comprises 21 items distributed in three subscales of seven items each, classifying severity into the following categories: normal, mild, moderate, severe, and extremely severe.

Bruxism Clinical Screening Self-Report Test: Bruxism was assessed using a screening instrument originally proposed by McNeill (1993) and later modified by Kindernecht (1997), in the version reported by De La Cruz and Quispe (2023) (22). The instrument comprises 25 dichotomous (Yes/No) items addressing symptoms and manifestations potentially related to bruxism across four domains: Interview (11 items), Tooth-level Damage (5 items), Muscle Disorders (5 items), and Joint Disorders (4 items). According to the scoring criterion specified for the instrument, a positive screening result was defined as two or more affirmative responses across the 25 items, whereas fewer than two affirmative responses constituted a negative screening result. Reliability was reported using Cronbach's alpha (*α*=0.821), and content validity was assessed through judgment by three experts (100%) according to De La Cruz and Quispe (2023) (22). For the purposes of the present study, participants with a positive screening result were classified as having suspected bruxism based on self-report, subsequently referred to as suspected bruxism. The terminology was interpreted in the context of the international consensus proposed by Lobbezoo et al. (2018), which distinguishes levels of diagnostic certainty according to the assessment methods employed (3). Given that the study did not include an independent standardized bruxism-specific clinical or instrumental assessment, the screening outcome was not classified as probable or clinically confirmed bruxism.

DMFT Index: Recording of decayed, missing, and filled teeth using an odontogram under the MINSA Technical Norm (23). Categories: None (DMFT=0), Low (1–6), Moderate (7–13), and High (≥14).

BEWE (Basic Erosive Wear Examination): Assessment of erosive wear by sextants (S1-S6), with a score of 0–3 per sextant and a total range of 0–18 (24, 25).

### 2.4 Procedure

The research protocol was first approved by the Faculty of Health Sciences of the National University Toribio Rodríguez de Mendoza of Amazonas on September 1, 2025. Subsequently, ethical approval was granted by the Institutional Research Ethics Committee (CIEI-UNTRM; code CIEI-N° 00258) on October 31, 2025. Authorization was then obtained from the National Penitentiary Institute (INPE), and participant recruitment and data collection commenced only after all institutional and ethical approvals had been obtained. No study procedures involving participants were initiated before institutional ethical approval had been obtained. Before any study procedure, the investigator explained the objectives of the study, answered participants' questions, obtained verbal informed consent approved by the ethics committee, and documented each participant's verbal informed consent in the study data collection log. Data collection was conducted in two phases. In the first phase, the principal investigator (M.J.T.), Bachelor in Psychology, supervised the administration of the DASS-21 and the Bruxism Clinical Screening Self-Report Test to each participant, remaining present during completion to clarify questions without influencing responses. In the second phase, the clinical evaluator (F.T.C.-Z.), Doctor Specialist in Stomatology and Orthodontics, performed the clinical oral examination of each participant, recording the DMFT caries index and BEWE by sextants. This oral examination was conducted separately from the self-report bruxism screening and was not used as an independent standardized clinical confirmation of bruxism. Before data collection, the examiner reviewed and standardized the diagnostic criteria for DMFT according to the MINSA Technical Norm and for BEWE according to Bartlett et al. (24). Both phases were conducted on the same day for each participant. All clinical oral examinations were performed by the same trained examiner, ensuring consistent application of the clinical criteria. Formal intra-examiner reproducibility, however, was not assessed.

### 2.5 Ethics statement

The study was approved by the Institutional Research Ethics Committee (CIEI) of the National University Toribio Rodríguez de Mendoza of Amazonas (UNTRM-A), code CIEI-N° 00258, dated October 31, 2025. Participant recruitment and data collection began only after ethical approval had been granted. Confidentiality was ensured through data anonymization. Participants provided verbal informed consent prior to evaluation. The verbal modality was justified by the inherent conditions of the penitentiary environment, where the signing of written documents may generate distrust and resistance to participation, thereby compromising the validity of responses. This modality was explicitly approved by the CIEI-UNTRM-A.

### 2.6 Data analysis

Normality of variables was assessed using the Shapiro–Wilk test (26), revealing non-normal distribution in all variables (*p* < 0.001). For bivariate analysis, the Chi-square test and Fisher's exact test were applied according to cell size (27). Prevalence estimates were reported with 95% confidence intervals (95% CIs) calculated using the Wilson method. Oral health analyzes were conducted using available data for each clinical indicator because complete clinical data were not available for all participants. Associations between suspected bruxism and ordinal psychological variables were additionally explored using Spearman's rank correlation coefficient (rho). Binary logistic regression was performed to explore independent associations with suspected bruxism. The dependent variable, suspected bruxism, was coded dichotomously (1 = present, 0 = absent). Independent variables included anxiety, depression, and stress (ordinal numerical variables from the DASS-21), sex (categorical: female = reference category, male = 1), and age group (ordinal numerical). Because exact ages were not available, the mean age was estimated from the midpoint of each predefined age category and is presented as a descriptive approximation. All prespecified variables were entered simultaneously into the model given the exploratory nature of the analysis. Multicollinearity among independent variables was assessed using the Variance Inflation Factor (VIF). Model goodness-of-fit was evaluated using the Hosmer-Lemeshow test, Nagelkerke R^2^ coefficient, and percentage of correct classification. Odds ratios (ORs) from the logistic regression model were reported with their corresponding 95% CIs. The significance threshold was *p* < 0.05. The tidyverse, gtsummary, flextable, and corrplot packages of R version 4.5.3 were used. For goodness-of-fit assessment, the ResourceSelection (Hosmer-Lemeshow) and DescTools (*N*agelkerke R^2^) packages were employed.

## 3 Results

### 3.1 General characteristics of the sample

A total of 201 persons deprived of liberty were evaluated. Males comprised 79.6% (*n* = 160) and females 20.4% (*n* = 41). Age was collected in predefined groups; descriptive statistics were estimated by assigning the midpoint of each interval (18–19 = 18.5; 20–29 = 24.5; 30–39 = 34.5; 40–49 = 44.5; ≥50 = 55.0 years). Using the midpoint of each age category, the estimated mean age was 38.6 years (SD = 10.5), with an approximate median of 34.5 years (range: 18 to ≥50 years). The most frequent age groups were 30–39 years and 40–49 years, both with 30.0% (*n* = 60 each). The majority presented normal levels across the three DASS-21 subscales: anxiety 79.1%, depression 73.0%, and stress 86.5% (Table 1).

**Table 1:** General characteristics of persons deprived of liberty from the huancas penitentiary establishment (*N* = 201).

Variable	n	%
Sex		
Female	41	20.4
Male	160	79.6
Age group		
18–19 years	2	1.0
20–29 years	44	22.0
30–39 years	60	30.0
40–49 years	60	30.0
≥ 50 years	34	17.0
Ward		
Women	41	20.4
Primary	73	36.3
Minimum security	87	43.3
Anxiety (DASS-21)		
Normal	159	79.1
Mild	12	6.0
Moderate	13	6.5
Severe	17	8.5
Depression (DASS-21)		
Normal	146	73.0
Mild	21	10.5
Moderate	20	10.0
Severe	3	1.5
Extremely severe	10	5.0
Stress (DASS-21)		
Normal	173	86.5
Mild	12	6.0
Moderate	9	4.5
Severe	4	2.0
Extremely severe	2	1.0
Suspected bruxism		
Present	146	72.6
Absent	55	27.4

DASS-21 = 21-item Depression, Anxiety and Stress Scale. Data are presented as absolute frequencies and percentages.

### 3.2 Prevalence of suspected bruxism according to sociodemographic characteristics

The overall prevalence of suspected bruxism was 72.6% (146/201; 95% CI: 66.1–78.3%). Prevalence was higher among women than men (80.5% vs. 70.6%), although the difference was not statistically significant (*p* = 0.20). By age group, prevalence was 80.4% among participants aged 18–29 years, 68.3% among those aged 30–49 years, and 76.5% among those aged ≥50 years, with no statistically significant differences between age groups (*p* = 0.60). By ward, the Women's ward showed the highest prevalence (80.5%; 95% CI: 66.0–89.8%), followed by Minimum Security (71.3%; 95% CI: 61.0–79.7%) and Primary Regime (69.9%; 95% CI: 58.6–79.2%), with no statistically significant differences between wards (*p* = 0.40) (Table 2, Figure 1).

**Table 2:** Prevalence of suspected bruxism according to sociodemographic characteristics (*N* = 201).

Variable	Total *n* (%)	No bruxism *n* (%)	Suspected bruxism *n* (%)	95% CI for suspected bruxism	p
Overall	201 (100)	55 (27.4)	146 (72.6)	66.1–78.3	-
Sex					0.20
Female	41 (20.4)	8 (19.5)	33 (80.5)	66.0–89.8	
Male	160 (79.6)	47 (29.4)	113 (70.6)	63.2–77.1	
Age group					0.60
18–29 years	46 (22.9)	9 (19.6)	37 (80.4)	66.8–89.3	
30–49 years	120 (59.7)	38 (31.7)	82 (68.3)	59.6–76.0	
≥ 50 years	34 (16.9)	8 (23.5)	26 (76.5)	60.0–87.6	
Ward					0.40
Women	41 (20.4)	8 (19.5)	33 (80.5)	66.0–89.8	
Primary	73 (36.3)	22 (30.1)	51 (69.9)	58.6–79.2	
Minimum security	87 (43.3)	25 (28.7)	62 (71.3)	61.0–79.7	

Data are expressed as *n* (%). Percentages in the Total column represent the proportion of the total study population, whereas percentages in the No bruxism and Suspected bruxism columns represent proportions within each sociodemographic subgroup. The 95% CIs correspond to the prevalence of suspected bruxism and were calculated using the Wilson method. Age data were available for 200 participants. Chi-square or Fisher's exact tests were applied as appropriate. *p* < 0.05 was considered statistically significant.

**Figure 1:**
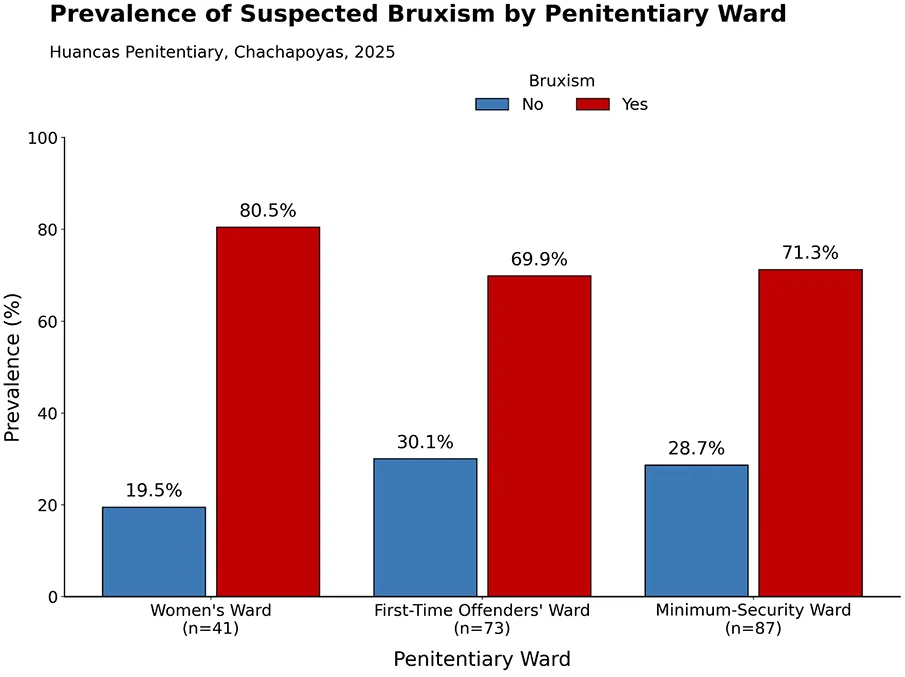
Prevalence onf suspected bruxism by penitentiary ward, huancas PE, 2025. Red bars indicate the proportion of persons deprived of liberty with suspected bruxism and blue bars indicate those without suspected bruxism, by ward.

### 3.3 Association between suspected bruxism and DASS-21

Anxiety severity showed a statistically significant overall association with suspected bruxism in the bivariate analysis (Fisher's exact test, *p* = 0.028). Severe anxiety was observed in 11.6% of participants with suspected bruxism and in none of those without suspected bruxism; however, this category-specific pattern should be interpreted descriptively because the *p*-value refers to the overall distribution of anxiety levels. No statistically significant overall associations were observed for depression (*p* = 0.12) or stress (*p* = 0.20), although moderate to extremely severe levels were more frequent among participants with suspected bruxism (Table 3, Figure 2).

**Table 3:** Levels of anxiety, depression, and stress (DASS-21) according to the presence of suspected bruxism (*N* = 201).

Variable	No bruxism *n* = 55 (%)	Suspected bruxism *n* = 146 (%)	p
Anxiety			0.028*
Normal	47 (85.5)	112 (76.7)	
Mild	4 (7.3)	8 (5.5)	
Moderate	4 (7.3)	9 (6.2)	
Severe	0 (0.0)	17 (11.6)	
Depression			0.12
Normal	46 (83.6)	100 (69.0)	
Mild	5 (9.1)	16 (11.0)	
Moderate	3 (5.5)	17 (11.7)	
Severe	1 (1.8)	2 (1.4)	
Extremely severe	0 (0.0)	10 (6.9)	
Stress			0.20
Normal	53 (96.4)	120 (82.8)	
Mild	1 (1.8)	11 (7.6)	
Moderate	1 (1.8)	8 (5.5)	
Severe	0 (0.0)	4 (2.8)	
Extremely severe	0 (0.0)	2 (1.4)	

Data are expressed as *n* (%). Fisher's exact test was applied. The reported *p*-values ​​correspond to overall comparisons across severity categories. *p < 0.05. DASS-21 = 21-item Depression, Anxiety and Stress Scale.

**Figure 2:**
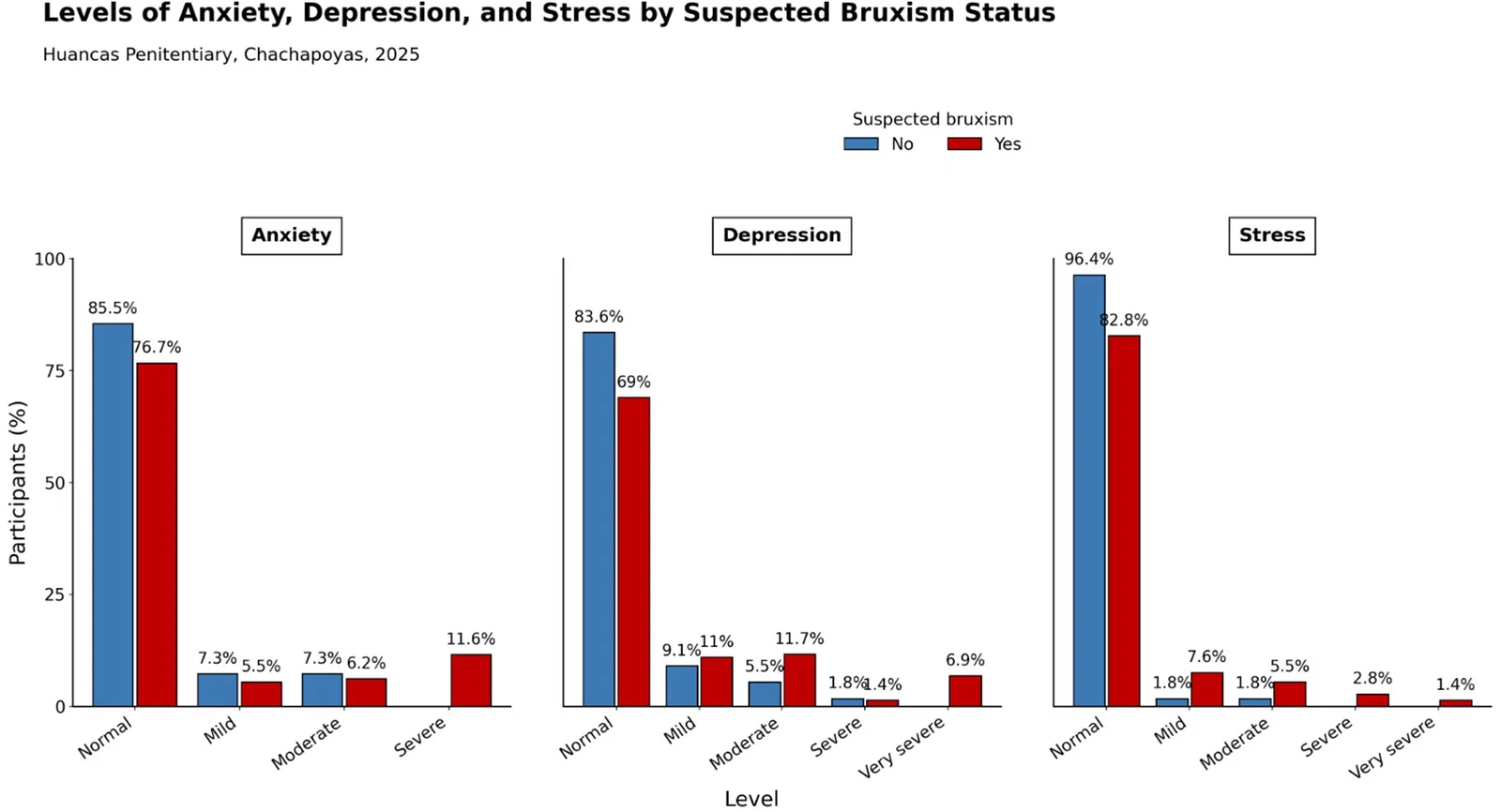
Levels of anxiety, depression, and stress (DASS-21) according to the presence of suspected bruxism, huancas PE, 2025. *Percentage distribution of DASS-21 severity levels among persons deprived of liberty with and without suspected bruxism. Red bars = suspected bruxism; blue bars = no suspected bruxism.*

### 3.4 Associations between suspected bruxism and DASS-21 scores

Spearman analysis showed weak positive associations between suspected bruxism and depression (rho = 0.159; *p* < 0.05) and between suspected bruxism and stress (rho = 0.179; *p* < 0.05). The association between suspected bruxism and anxiety was also weak but not statistically significant (rho = 0.108; *p* > 0.05). Given the small magnitude of these coefficients and the dichotomous nature of the suspected bruxism variable, these findings should be interpreted as weak associations rather than evidence of a strong relationship. The DASS-21 subscales were strongly correlated with each other (rho = 0.70–0.80; *p* < 0.05), indicating substantial overlap among the psychological dimensions (Table 4, Figure 3).

**Table 4:** Spearman associations between DASS-21 subscales and suspected bruxism.

Variable	Anxiety	Depression	Stress	Suspected bruxism
Anxiety	1.00	0.772*	0.803*	0.108
Depression	0.772*	1.00	0.705*	0.159*
Stress	0.803*	0.705*	1.00	0.179*
Suspected bruxism	0.108	0.159*	0.179*	1.00

Values ​​represent Spearman's rho coefficients. Associations between suspected bruxism and DASS-21 subscales should be interpreted considering the dichotomous nature of the suspected bruxism variable and the small magnitude of the observed coefficients. *p < 0.05. DASS-21 = 21-item Depression, Anxiety and Stress Scale.

**Figure 3:**
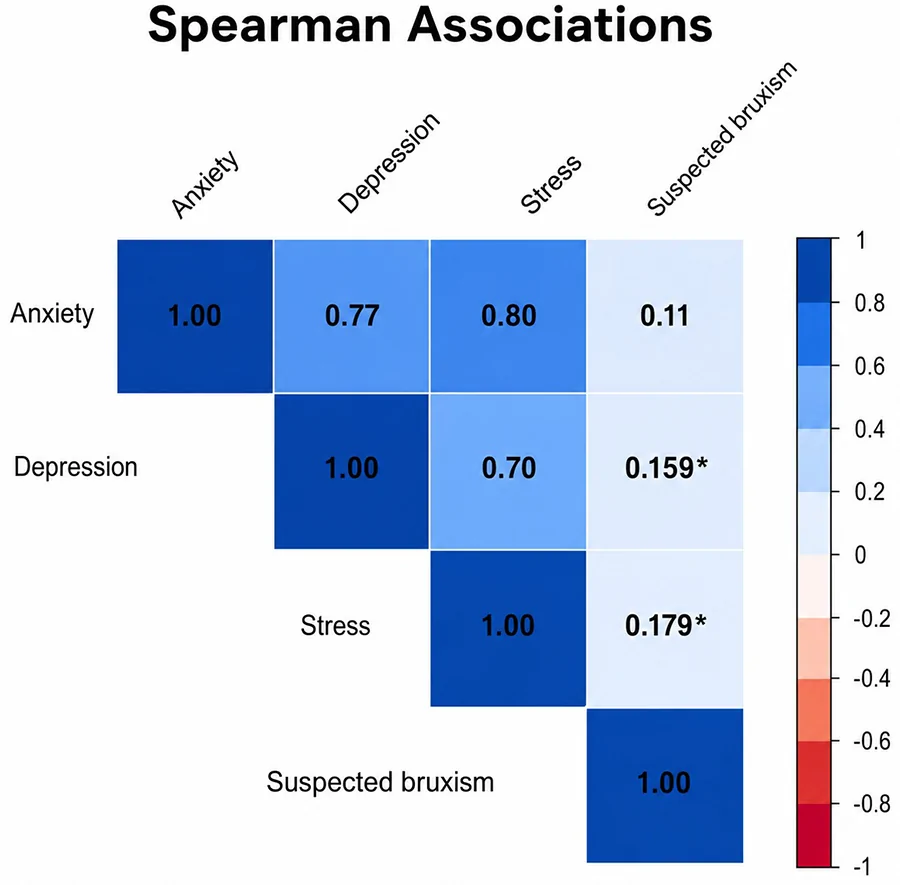
Spearman association matrix between psychological variables and suspected bruxism, huancas PE, 2025. Color intensity reflects the magnitude of the Spearman correlation coefficient. Values ​​represent Spearman's rho. Asterisks indicate statistically significant associations (p < 0.05).

### 3.5 Binary logistic regression

No statistically significant independent associations with suspected bruxism were identified in the logistic regression model (all *p* > 0.05). Stress showed the largest odds ratio (OR = 2.51; 95% CI: 0.64–17.0; *p* = 0.30), although the estimate was imprecise and not statistically significant (Table 5). The Nagelkerke R^2^ was 0.066, indicating limited explanatory capacity of the model. The Hosmer-Lemeshow test was significant (Chi^2^ = 18.82; df = 8; *p* = 0.016), indicating unsatisfactory model calibration and warranting caution in the interpretation of the multivariate estimates. The percentage of correct classification was 72.4%; however, this value should be interpreted cautiously because of the high baseline prevalence of suspected bruxism. The lack of statistically significant independent associations should also be interpreted cautiously in view of the substantial overlap among the DASS-21 subscales (rho = 0.70–0.80; maximum VIF for anxiety = 7.10), sparse observations in some response categories, and wide confidence intervals, all of which may have reduced the precision and stability of the regression estimates.

**Table 5:** Binary logistic regression of factors associated with suspected bruxism among persons deprived of liberty (*N* = 201).

Variable	OR	95% CI	p
Anxiety (DASS-21)	0.83	0.28–2.75	0.70
Depression (DASS-21)	1.41	0.75–3.10	0.30
Stress (DASS-21)	2.51	0.64–17.0	0.30
Sex (male vs. female)	1.30	0.30–5.97	0.70
Age group	0.93	0.67–1.27	0.60

OR = Odds Ratio; 95% CI = 95% Confidence Interval. Dependent variable: suspected bruxism (1 = present, 0 = absent). Reference category for sex: Female. Nagelkerke R^2^ = 0.066; Hosmer-Lemeshow Chi^2^ = 18.82, df = 8, *p* = 0.016; correct classification = 72.4%.

### 3.6 Oral health status

Among participants with available DMFT data, 81.1% (90/111) were classified in the high caries category, with no statistically significant difference in DMFT categories according to suspected bruxism status (*p* = 0.30). The mean BEWE score was 8.00 ± 6.57, with no statistically significant difference according to suspected bruxism status (*p* = 0.40) (Table 6, Figure 4).

**Table 6:** Oral health status according to suspected bruxism.

Variable	No suspected bruxism *n* = 55	Suspected bruxism *n* = 146	p
DMFT caries index	*n* = 27	*n* = 84	0.30
Low	3 (11.1%)	3 (3.6%)	
Moderate	3 (11.1%)	12 (14.3%)	
High	21 (77.8%)	69 (82.1%)	
BEWE total, mean ± SD	8.47 ± 6.04	7.83 ± 6.77	0.40

Data are presented for participants with available information for each oral health indicator. For the DMFT caries index, data were available for 111 participants (27 without suspected bruxism and 84 with suspected bruxism). Percentages were calculated using the number of participants with available data within each suspected bruxism group as the denominator. BEWE = Basic Erosive Wear Examination; SD = standard deviation. Fisher's exact test was used for categorical variables and the Wilcoxon rank-sum test for BEWE.

**Figure 4:**
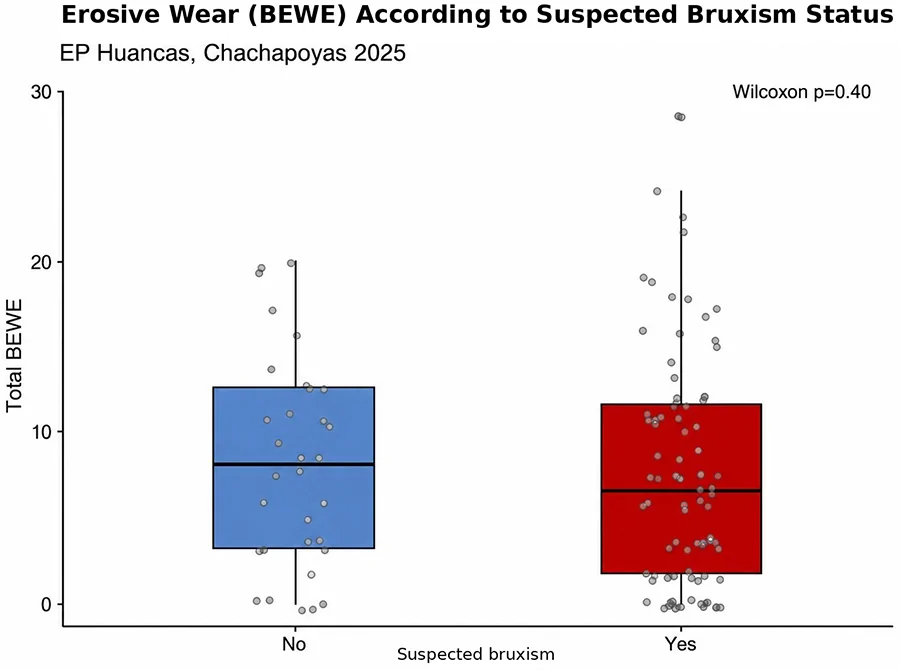
Distribution of total BEWE score according to suspected bruxism status, huancas PE, 2025. Box plots show the distribution of total BEWE scores, with individual observations displayed as points. The line within each box represents the median. No statistically significant difference was observed between participants with and without suspected bruxism (Wilcoxon test, *p* = 0.40).

## 4 Discussion

The most relevant finding of the present study is the high prevalence of screening-defined suspected bruxism (72.6%) among persons deprived of liberty at the Huancas Penitentiary Establishment. This estimate exceeds the prevalence reported in adults from the general population, ranging from 8% to 31.4% according to recent systematic reviews and meta-analyses (1–3). A systematic review on bruxism epidemiology in Latin America between 2019 and 2024 reported a prevalence of 41.1% in Brazil as the highest in the region, while studies conducted in Peruvian university populations reported prevalence ranging from 39.6% to 69.2%. However, these comparisons should be interpreted cautiously because prevalence estimates of bruxism are highly dependent on the assessment method, operational definition, and diagnostic criteria applied across studies. In the present study, suspected bruxism was identified using a self-report screening instrument rather than an independent standardized bruxism-specific clinical or instrumental assessment. Therefore, the high prevalence observed may partly reflect differences in measurement and classification procedures and cannot be directly interpreted as evidence of a higher prevalence of clinically confirmed bruxism in this prison population. Although psychosocial stress and other psychological factors have been associated with bruxism in previous studies (6, 7, 11, 13), the cross-sectional design and screening-based outcome used in the present study do not allow the observed prevalence to be attributed to the penitentiary environment.

Although the difference was not statistically significant, the higher prevalence of suspected bruxism observed in the women's pavilion (80.5%) is consistent with previous reports suggesting a greater prevalence of bruxism among women, possibly mediated by sex-related differences in the neuroendocrine response to stress (1, 28–30). Various authors have suggested that this difference could be related to variations in neuroendocrine response to stress, as well as a higher prevalence of anxiety and affective symptoms in women (31, 32). Although the trend observed in our study coincides with available literature, the difference between sexes did not reach statistical significance, likely due to the reduced sample size of the female group (*n* = 41).

The bivariate analysis showed a statistically significant association between anxiety severity and suspected bruxism (Fisher's exact test, *p* = 0.028). Notably, severe anxiety was observed only among participants with suspected bruxism, although this finding should be interpreted cautiously given the small number of individuals in this category. This pattern is consistent with neurobiological models proposing that anxiety is associated with hyperactivity of the autonomic nervous system and the hypothalamic-pituitary-adrenal axis, linked to mandibular parafunctional muscle activity (28, 33–35). Likewise, recent systematic reviews have documented consistent associations between bruxism, anxiety, stress, and other psychological factors in both the general population and clinical groups (1, 6).

The weak positive associations observed between suspected bruxism and depression (rho=0.159; *p* < 0.05) and between suspected bruxism and stress (rho=0.179; *p* < 0.05) should be interpreted cautiously given their small magnitude. Regarding depression, the systematic review by Belenda González et al. confirmed the existence of an association between bruxism and depressive symptoms in adults, suggesting shared neurobiological mechanisms involving dysregulation of the dopaminergic and serotonergic systems (6). Smardz et al., in a polysomnographic study, found significant correlation between sleep bruxism, stress, and depression (28). In our study, 5.0% of participants presented extremely severe depression, and these cases occurred exclusively among those with suspected bruxism; however, the small number of observations in this category warrants cautious interpretation. Regarding stress, neurobiological literature proposes that chronic activation of the hypothalamic-pituitary-adrenal axis elevates cortisol levels, which is associated with hypertonicity of masticatory muscles (33, 34). The meta-analysis by Chemelo et al. confirmed that individuals with stress have a significantly higher probability of presenting bruxism (7). In the penitentiary context, factors such as overcrowding, loss of autonomy, and forced cohabitation have been associated with psychological distress and may contribute to a stressful environment (11, 13). Although both associations were statistically significant, their weak magnitude indicates that the observed relationships were small and should not be overinterpreted (6). In contrast, none of the psychological variables showed statistically significant independent associations with suspected bruxism in the multivariable model. This finding should be interpreted cautiously, as moderate multicollinearity among the DASS-21 subscales and sparse observations in some response categories may have reduced the precision of the regression estimates, thereby limiting the ability to identify independent associations.

The penitentiary context as a risk factor for mental disorders is well-documented. Favril et al. in The Lancet Public Health confirmed the high burden of mental illnesses in persons deprived of liberty worldwide (11). González-Riera et al. documented increases in stress, anxiety, and depression among inmates during periods of isolation (13). These contextual factors may contribute to psychological distress among persons deprived of liberty; however, their specific relationship with suspected bruxism could not be established in the present study.

Among participants with available DMFT data, 81.1% were classified in the high caries category, consistent with research in other penitentiary contexts documenting a high caries burden and unmet dental needs (14–16, 18). In the same penitentiary establishment, previous research also reported a generalized burden of untreated oral disease, particularly edentulism, reflecting the persistent limitations in access to dental care over time (17). No statistically significant difference in BEWE scores was observed according to suspected bruxism status (*p* = 0.40). Because the present study did not specifically assess the determinants of erosive tooth wear, its etiology cannot be inferred from these findings. The review by Amaya et al. highlighted the global scarcity of dental services in penitentiary establishments (19).

The coexistence of a high prevalence of screening-defined suspected bruxism, psychological symptoms, and poor oral health highlights the potential relevance of considering oral and mental health together within correctional healthcare. Integrated dental–psychological screening approaches combining validated psychological instruments with routine oral examinations could represent a potential strategy for identifying individuals who may benefit from further assessment. In this context, dental professionals may provide an additional opportunity to identify individuals with oral findings and psychological symptoms who could require further psychological assessment and referral. However, the effectiveness and feasibility of such integrated approaches were not evaluated in the present study and should be investigated in future intervention and implementation studies.

The main limitations of this study include its cross-sectional design, which precludes causal inference; the inclusion of participants from a single penitentiary establishment, which may limit the generalizability of the findings; the exclusion of individuals receiving psychotropic medication, which may have reduced the representation of persons receiving treatment for psychiatric conditions, potentially limiting the generalizability of the observed associations between psychological symptoms and suspected bruxism; the use of self-report instruments for the assessment of psychological symptoms and suspected bruxism, which may be susceptible to reporting and social desirability bias (36); and the absence of information on potentially relevant variables such as duration of incarceration, type of offense, and substance use. The lack of information on incarceration duration is especially relevant because prolonged exposure to the prison environment may influence both psychological distress and parafunctional behaviors, potentially acting as an unmeasured confounding factor. In addition, suspected bruxism was identified using a self-report screening instrument without independent standardized bruxism-specific clinical or instrumental confirmation. Therefore, the outcome should be interpreted as screening-defined suspected bruxism rather than clinically confirmed or probable bruxism according to the diagnostic certainty framework proposed in the international consensus (3). The screening approach also did not allow awake and sleep bruxism to be evaluated separately. Age was recorded in categories rather than exact years, limiting the precision of descriptive age estimates. All clinical oral examinations were performed by a single trained examiner. Although this avoided inter-examiner variability, formal intra-examiner reproducibility was not assessed through repeated examinations; therefore, intra-examiner agreement statistics were not available for the DMFT and BEWE assessments. This should be considered when interpreting the clinical oral health findings. In addition, complete oral health data were not available for all participants, resulting in variable denominators across the clinical oral health analyzes and limiting the precision and representativeness of these secondary findings. Finally, some psychological symptom categories included few participants, and moderate multicollinearity was observed among the DASS-21 subscales, which, together with the unsatisfactory calibration of the logistic regression model, may have reduced the precision and stability of the multivariate estimates despite the inclusion of the full eligible prison census. Despite these limitations, this study provides novel evidence on the simultaneous burden of screening-defined suspected bruxism, psychological symptoms, and poor oral health among persons deprived of liberty in the Peruvian Amazon, a population that remains largely underrepresented in the scientific literature.

## 5 Conclusions

The prevalence of screening-defined suspected bruxism among persons deprived of liberty in the Huancas Penitentiary Establishment was high (72.6%), although direct comparisons with estimates from other populations should be made cautiously because of differences in assessment methods and diagnostic criteria. Anxiety severity showed a significant overall bivariate association with suspected bruxism, with severe anxiety observed only among participants with suspected bruxism, although this category-specific pattern should be interpreted cautiously. Depression and stress showed weak but statistically significant positive associations with suspected bruxism. In addition, the oral health findings indicated a considerable burden of dental disease among participants with available clinical data, although no statistically significant differences in DMFT categories or BEWE scores were observed according to suspected bruxism status.

Taken together, these findings indicate the coexistence of screening-defined suspected bruxism, psychological symptoms, and poor oral health in this prison population and highlight the potential value of considering oral and mental health jointly within correctional healthcare. Integrated dental–psychological assessment may provide an opportunity to identify individuals who could benefit from further evaluation and referral, with dental professionals potentially serving as an additional point of contact within correctional healthcare services. However, the feasibility and effectiveness of integrated screening and multidisciplinary care were not evaluated in this study and should be examined in future prospective and intervention studies. Further research using standardized bruxism-specific clinical or instrumental assessment is also warranted to confirm these findings and better characterize the relationship between bruxism and psychological symptoms among persons deprived of liberty.

## Data Availability

The raw data supporting the conclusions of this article will be made available by the authors, without undue reservation.
